# Trace elements in end-stage renal disease – unfamiliar territory to be revealed

**DOI:** 10.1186/1471-2369-10-12

**Published:** 2009-06-02

**Authors:** Adrian Covic, Paul Gusbeth-Tatomir

**Affiliations:** 1 Nephrology Clinic, Parhon University Hospital, "Gr.T. Popa" University of Medicine and Pharmacy, Iasi, Romania

## Abstract

Although associated with unfavorable outcomes in the general population, abnormal blood levels of various trace elements have not been consistently studied in the end-stage renal disease population (with the notable exception of aluminum). This is surprising, as the uremic patient treated by chronic dialysis loses one major route of trace element excretion and is exposed systematically to a foreign environment (the dialysis fluid) possibly contaminated with significant amounts of potential deleterious trace elements. Moreover, some biological important trace elements may be lost through the dialysis membrane. Most studies to date demonstrated significantly altered blood levels of trace elements in ESRD patients compared to healthy controls. However, the biological impact of these abnormalities in renal disease is largely unknown and should be clarified by future studies. A further step would be the design of well-controlled randomized interventional studies, examining the potential therapeutic benefit of supplementing one or more trace elements in ESRD patients, a population characterized by an impressive mortality due to cardiovascular, infectious and neoplasic disease.

## 

End-stage renal disease (ESRD) patients have a mortality risk several times higher that their counterparts without significant renal disease. This is due mainly to cardiovascular (CV) disease, but is also caused by the enhanced risk for infectious diseases and neoplasia [1]. Despite spectacular improvements in chronic renal replacement therapies (RRT), this excessive mortality remains particularly high in dialysis patients, compared to the general population. Extensive work has been done in the last decade on the traditional and non-traditional CV risk factors in ESRD. Research has been focused on the impact of "mineral" (namely calcium and phosphate metabolism) abnormalities and iron store derangements on morbidity and mortality in ESRD patients [2,3]. This is not surprising, as this work was largely driven by the industry, highly interested in promoting drugs claimed to correct anemia or calcium-phosphate metabolism abnormalities in a growing dialysis population.

Although derangements in the metabolism of several trace elements such as antimony, arsenic cadmium, molybdenum, nickel, selenium and so on have been reported [4] for several decades in patients with chronically (severely) reduced renal function, such work – not sustained by powerful companies and rather anecdotal, has been largely ignored by the nephrological community. One could speculate that the "trace" character of these elements discouraged more extensive research for psychological reasons. Trace elements are not (yet) routinely determined in the blood of dialysis patients and many uncertainties regarding these substances may trigger reluctance in the mind of the practicing nephrologists.

Therefore, the systematic review and meta-analysis published by Tonelli and co-workers this month in *BMC Medicine *[5] may be of particular relevance. The authors identified 128 – mostly European – eligible studies examining the whole blood, plasma, or serum levels of nineteen trace elements in chronic hemodialysis patients (HD – the main modality of chronic RRT) in comparison to healthy subjects. Overall, available literature suggests that the blood levels of some trace elements such as cadmium, chromium, fluorine, iodine, lead, or vanadium are high in ESRD, whereas the levels of selenium, zinc or manganese are lower in HD patients, compared to controls. The magnitude of these differences was pronounced, suggesting that renal patients could have subsequent different but relevant (albeit in many cases asymptomatic) clinical characteristics in this respect. Moreover, according to the authors' investigation, the blood concentration of antimony, iodine (!), tellurium, and thallium in renal failure patients are a *terra incognita *for research until yet. The authors conclude that, according to their review and meta-analysis, the average blood levels for several (potentially) biologically important trace elements are different in hemodialysis patients compared to healthy control subjects. This is not necessarily surprising, as more familiar issues such as total cholesterol or blood pressure have a peculiar significance in ESRD patients compared to the general population; this phenomenon is generally characterized as "inverse epidemiology" [6].

During the HD session, low-weight (and some middle weight) molecules are removed driven by concentration and pressure gradients through a semi-permeable membrane. These processes are essential for the survival of ESRD patients, as the dialysis process substitutes, far from perfectly, for the excretory functions of the kidneys. However, in contrast to native kidneys, this depuration process is non-discriminatory. As the authors highlight in their article, hemodialysis may lead to a depletion of some biologically essential substances, possibly already deficient due to dietary restrictions. Moreover, trace substances present in the dialysate may switch over to the blood and accumulate in the patient, due to the lack of clearance by the dialysis membrane and the (potential) remnant renal function. This possibility was a harsh lesson for nephrologists more than two decades ago, when it became obvious that the (very efficient!) phosphate binder aluminum hydroxide (as well as the contamination of the dialysate water with aluminum) may lead to severe complications such as aluminum dementia, severe anemia or adynamic bone disease in HD patients [7].

Low levels of some essential trace elements have been associated with adverse outcome in the general population. For instance, zinc deficiency is linked to delayed wound healing and decreased immunity leading to increased infection susceptibility [8], frequently encountered in uremic patients. Moreover, excess concentrations of cadmium and arsenic, as well as selenium deficiency, have been associated with carcinogenicity in non-renal populations [9]. Highly speculative (but pathophysiologically credible), reduced or enhanced blood levels of some trace elements may be (even more) deleterious for the chronic HD population.

The main merit of the discussed paper is that it offers the first comprehensive and statistically very accurate review and meta-analysis on trace elements blood levels in HD patients compared to healthy controls. Of note however, as the authors pointed out [5], matching for age and gender was poor in the studies examined, eligibility status and measurement error assessment were infrequent, although technique was generally well-reported. Even a mandatory issue such as the time spent on dialysis was infrequently reported, although clearly significant for depletion or accumulation of some trace elements. This is not surprisingly, given the "Cinderella-status" of most of these reports. Moreover, clinical studies in nephrology and particularly in ESRD patients are generally under-powered, far from the statistically accuracy of investigations in larger populations [10].

The key point is that the *biological significance *of either accumulation or depletion of the studied trace elements in the blood is largely unknown, at least in uremic patients. Some data are available, on rather historical grounds, on lead and arsenic accumulation [4,11]. The scarcity of information is surprising, as the hemodialysis patient is largely (and repeatedly) exposed to a "foreign" medium which is the dialysate fluid. This water used for dialysis is checked, according to current national and international regulations, for some but not all relevant trace elements. As mentioned, the measurement of blood levels of trace elements is unusual in the clinical practice of dialysis units, whereas the behavior of elements or molecules such as sodium, potassium, calcium, glucose or bicarbonate are well-studied [12,13].

A serious limitation of the analyzed studies is that these focus their investigation on the uremic blood. Moreover, in some studies, whole blood concentrations of trace elements are investigated, whereas in others, just the serum or the plasma levels of such elements are analyzed. However, the physiology of trace elements is far more complicated, implying various routes of excretion, protein binding and tissue concentrations [14]. Tissue concentrations of trace elements may markedly differ from blood, serum or plasma concentrations. Furthermore, the sequestration of trace elements is different from organ to organ, as shown by studies investigating tissue deposition of aluminum or lanthanum [7,15].

The excellent review and meta-analysis of Tonelli et al [5] is first of all a call for a more serious and systematic investigation of the role of trace elements disturbances in uremic patients on hemodialysis. It is obvious that ESRD have other characteristics in respect of trace elements than the general population. Their correlates to infectious, cardiovascular and neoplasic complications should be extensively studied, as well as the potential interventions. Given the actual therapeutic *nihilism *in renal patients [10] this is not an easy task. However, well-controlled studies on the clinical impact of trace elements blood and tissue levels in ESRD are mandatory. In addition, intervention studies using trace elements supplementation would be even more helpful for the fate of the ESRD patient.

## Competing interests

Professor Adrian Covic received consultant fees from Fresenius Medicale Care Romania.

## Pre-publication history

The pre-publication history for this paper can be accessed here:

http://www.biomedcentral.com/1471-2369/10/12/prepub
